# Publisher Correction: Thermo-optically induced transparency on a photonic chip

**DOI:** 10.1038/s41377-021-00695-3

**Published:** 2021-12-29

**Authors:** Marco Clementi, Simone Iadanza, Sebastian A. Schulz, Giulia Urbinati, Dario Gerace, Liam O’Faloain, Matteo Galli

**Affiliations:** 1 Dipartimento di Fisica, Università di Pavia, Via A. Bassi 6, 27100 Pavia, Italy; 2 Centre for Advanced Photonics and Process Analysis, Munster Technological University, Rossa Ave Bishopstown, Cork, T12 P928 Ireland; 3 Tyndall National Institute, Lee Maltings Complex Dyke Parade, Cork, T12 R5CP Ireland; 4 SUPA, School of Physics and Astronomy, University of St. Andrews, Fife, KY16 9SS UK; 5 Present Address: École Polytechnique Fédérale de Lausanne, Photonic Systems Laboratory (PHOSL), STI-IEL, Station 11, Lausanne, 1015 Switzerland

**Keywords:** Nanophotonics and plasmonics, Slow light

Correction to: *Light: Science & Applications*

10.1038/s41377-021-00678-4 published online 3 December 2021

Following the publication of this article^1^, it is noticed this article contained some errors. Details are listed below.

1) The letter ‘c’ in front of Eq. (1a) should be removed. The correct Eq. (1a) should be:

1a $$\frac{d}{{dt}}a\left( t \right) = \left( {i\omega _0 - \frac{\Gamma }{2}} \right)a\left( t \right) + iG\Delta T\left( t \right)a\left( t \right) + \sqrt {\eta \Gamma } s_{in}\left( t \right)$$

2) In Fig. 1a, left panel: The Greek letter “alpha” should be corrected to the letter ‘a’. Correct fig. 1a is included in this Correction.

**Figure 1:**
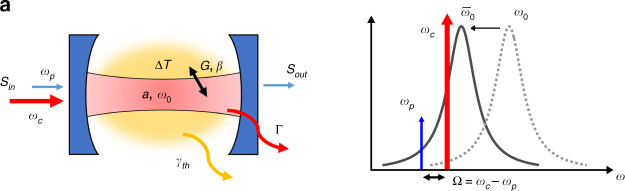
Fig. 1

3) Reference 15 need to be changed to below: M. Clementi, M. Galli, L. O’Faolain, and D. Gerace, “Electromagnetically induced transparency from first-order dynamical systems,” Phys. Rev. B 104, 205434 (2021).

The original article has been updated.
