# Supplementary material for: Thermo-optically induced transparency on a photonic chip
Source: Light Sci Appl. 2021 Dec 3;10:240. doi: 10.1038/s41377-021-00678-4 (PMC8642398; doi:10.1038/s41377-021-00678-4)
Supplement: Supplementary file 1 — Supplementary online material [file 41377_2021_678_MOESM1_ESM.pdf]

Supplementary Online Material for: Thermo-Optically Induced Transparency on a photonic chip

Marco Clementi,[1*] Simone Iadanza,[2,3] Sebastian A. Schulz,[4]
Giulia Urbinati,[1] Dario Gerace,[1] Liam O'Faloain,[2,3] Matteo Galli[1*]

[1]Dipartimento di Fisica, Università di Pavia, Via A. Bassi 6, 27100 Pavia, Italy

[2]Centre for Advanced Photonics and Process Analysis, Munster Technological University, Rossa Ave Bishopstown, Cork T12 P928, Ireland

[3]Photonics, Tyndall National Institute, Lee Maltings Complex Dyke Parade, Cork T12 R5CP, Ireland

[4]SUPA, School of Physics and Astronomy, University of St. Andrews, Fife KY16 9SS, UK

*Corresponding authors: marco.clementi01@universitadipavia.it, matteo.galli@unipv.it

Contents

S1 General model **S3**

S1.1 Driving field S5

S1.2 Steady state solution for the average pump field S5

S1.3 Linearized equations of motion S7

S1.4 Analytic solutions for the field amplitudes S8

S1.5 Output power (optical beat) S9

S2 Modelling multiple thermal decay rates **S14**

S2.1 Steady state solution S16

S2.2 Dynamical solution S16

S3 Experimental methods **S20**

S3.1 Sample design and fabrication S20

S3.2 Experimental setup S20

S3.3 Coupled-mode theory for the resonant scattering apparatus S22

S3.4 Static sample characterization S23

S4 Switch-on measurements **S26**

S5 Optical heterodyne measurements **S28**

S6 Engineering the thermal response **S31**

S7 Measurements dataset **S34**

S1 General model

Consider a lossy optical resonator, characterized by linear and nonlinear absorption, and affected by thermo-optical interaction. In the formalism of input-output relations (*1, 2*), this can be described by the following equations:

$$\frac{da(t)}{dt} = \left(i\omega_0 - \frac{\Gamma(U)}{2}\right) a(t) + i\omega_0 \alpha \Delta T(t) a(t) + \sqrt{\eta\Gamma(U)} s_{\text{in}}(t) \tag{S1a}$$

$$C_p \frac{d(\Delta T)}{dt} = \overline{\Gamma}_{\text{abs}}(U)|a(t)|^2 - K\Delta T(t) \tag{S1b}$$

The first expression is a dynamic equation for the field amplitude, which can be derived as a classical equation of motion from the quantized Hamiltonian $\hat{\mathcal{H}} = \hbar\omega_0 (1 + \alpha\Delta T) \left(\hat{a}^\dagger \hat{a} + \frac{1}{2}\right) + i\hbar\sqrt{\eta\Gamma} \left(s_{\text{in}}\hat{a}^\dagger - s_{\text{in}}^* \hat{a}\right)$, which depends parametrically on ΔT, and where $\hat{a}^\dagger$ ($\hat{a}$) is the suitably defined creation (annihilation) operator of the single-mode electromagnetic excitations in the resonator (*2*). Here:

- $a(t)$ is the modal field amplitude, normalized such that $U = |a(t)|^2$ is the mode energy;
- ω_0 is the bare resonance frequency;
- $\Gamma(U)$ is the mode decay rate, which depends on the mode energy U owing to nonlinear effects (two-photon absorption, TPA, free-carrier absorption, FCA), according with the definition $\Gamma(U) = \Gamma_{\text{rad}} + \Gamma_{\text{abs}} + \Gamma_{\text{TPA}}(U) + \Gamma_{\text{FCA}}(U)$;
- $s_{\text{in}}(t)$ is the driving field, normalized such that $|s_{\text{in}}(t)|^2$ is the input power;
- $\eta = \eta_{\text{in}} = \Gamma_{\text{ext}}^{\text{in}}/\Gamma(U)$ is the coupling efficiency, given an in-coupling rate $\Gamma_{\text{ext}}^{\text{in}}$.

- α is the thermo-optic coefficient, according with the empiric relation $\overline{\omega}_0 = \omega_0 + \omega_0 \alpha \Delta T$, and it is negative for silicon at telecom wavelengths.

The second expression is a rate equation that models the thermal dynamics, where:

- ΔT is an effective temperature offset (with respect to room temperature);
- $\overline{\Gamma}_{\text{abs}}(U)$ represents the (linear and nonlinear) absorption rate for the mode energy, which contributes to heat the cavity, according with the relation $\overline{\Gamma}_{\text{abs}}(U) = \Gamma_{\text{abs}} + \Gamma_{\text{TPA}}(U) + \Gamma_{\text{FCA}}(U)$;
- C_p is an effective heat capacity;
- K is an effective (extensive) heat conductivity.

Equations (S1) are nonlinear and mutually coupled via the dynamic variables $a(t)$ and $\Delta T(t)$. In particular, owing to nonlinear absorption effects[1], the cavity decay rate $\Gamma(U)$ and the optical absorption rate $\overline{\Gamma}_{\text{abs}}(U)$ depend explicitly on the cavity energy. We will limit ourselves for simplicity to the case where linear absorption only contributes to heating, while the cavity decay rate depends on both linear absorption (Γ_{abs}) and out-of-plane scattering (Γ_{rad}). This approximation is well justified provided that $\Gamma_{\text{abs}}/\overline{\Gamma}_{\text{abs}}(U) \approx 1$, namely the parasitic absorption from TPA and FCA is low compared to linear absorption. In principle, in the presence of important nonlinear absorption originated by a strong driving field, the total absorption loss $\overline{\Gamma}_{\text{abs}}(U)$ can be anyhow modeled as a static contribution, upon which the problem is linearized.

Under these assumptions, we define the parameters $G = \alpha\omega_0$, $\beta = \Gamma_{\text{abs}}/C_p$ and $\gamma_{\text{th}} =$

[1]We are neglecting the dispersive contributions from free-carrier dispersion and Kerr effects, which are negligible with respect to thermo-optic effect in this context.

K/C_p and we rewrite:

$$\frac{da(t)}{dt} = \left(i\omega_0 - \frac{\Gamma}{2}\right) a(t) + iG\Delta T(t)a(t) + \sqrt{\eta\Gamma}s_{\rm in}(t) \tag{S2a}$$

$$\frac{d(\Delta T)}{dt} = \beta|a(t)|^2 - \gamma_{\rm th}\Delta T(t) \tag{S2b}$$

S1.1 Driving field

We will assume the driving term in the form:

$$s_{\rm in}(t) = \overline{s}_{\rm in}e^{+i\omega_{\rm c}t} + \delta s_{\rm in}(t)$$

where $\delta s_{\rm in}(t) = s_{\rm p}e^{+i(\omega_{\rm c}-\Omega)t}$. The expression represents an input signal composed by an intense pump (control field), oscillating at frequency $\omega_{\rm c}$, and a weak probe, oscillating at frequency $\omega_{\rm p} = \omega_{\rm c} - \Omega$, and characterized by an amplitude $s_{\rm p}$, such that $|s_{\rm p}|^2 \ll |\overline{s}_{\rm in}|^2$. Upon this assumption, we will consider the thermal and optical response of the cavity as a constant value added to a small perturbation:

$$a(t) = \overline{a}e^{+i\omega_{\rm c}t} + \delta a(t) \tag{S3a}$$

$$\Delta T(t) = \overline{\Delta T} + \delta T(t) \tag{S3b}$$

S1.2 Steady state solution for the average pump field

By temporarily setting $\delta s_{\rm in} = 0$, we derive the equilibrium state for the pump field. We re-write Eqs. (S2):

$$
\begin{aligned}
i\omega_c\overline{a} &= \left(i\omega_0 - \frac{\Gamma}{2}\right)\overline{a} + iG\overline{\Delta T}\overline{a} + \sqrt{\eta\Gamma}\overline{s}_{\mathrm{in}} \\
0 &= \beta|\overline{a}|^2 - \gamma_{\mathrm{th}}\overline{\Delta T}
\end{aligned}
$$

which provide the steady state solutions:

$$
\overline{a} = \frac{\sqrt{\eta\Gamma}}{i\overline{\Delta} + \Gamma/2}\overline{s}_{\mathrm{in}} \qquad \overline{\Delta T} = \frac{\beta}{\gamma_{\mathrm{th}}}|\overline{a}|^2 \tag{S4}
$$

where $\overline{\Delta} = \omega_c - \overline{\omega}_0 = \omega_c - \left(\omega_0 + G\overline{\Delta T}\right)$ is the pump-cavity detuning in the presence of static thermo-optic shift and $|\overline{a}|^2$ represents the intracavity energy in the presence of a constant driving field.

By strict analogy with the theory of bistability for a localized mode coupled to a Kerr medium (*3*), Eq. (S4) can be re-written in the form:

$$
\frac{|\overline{a}|^2}{|\overline{s}_{\mathrm{in}}|^2} = \frac{4\eta/\Gamma}{1 + \left(2\Delta/\Gamma + |\overline{a}|^2/|\overline{a}_{\mathrm{b}}|^2\right)^2} \tag{S5}
$$

where we have defined the characteristic bistability energy $|\overline{a}_{\mathrm{b}}|^2 = -\frac{K\Gamma}{2G\Gamma_{\mathrm{abs}}}$. Remarkably, even in the absence of nonlinear absorption, Eqs. (S4) have a bistable solution for appropriate driving conditions, namely for $\Delta > \frac{\sqrt{3}}{2}\Gamma$. Under such conditions, one of the two stable equilibrium solutions should be appropriately chosen (*4*). As it will be shown in the following, the quantity $|\overline{a}_{\mathrm{b}}|^2$ has a crucial role in the interpretation of TOIT.

S1.3 Linearized equations of motion

The weak probe field can then be included in the model by linearizing the equations of motion (S2) at the equilibrium point, i.e., by inserting the dynamical variables (S3) into the coupled differential equations and getting:

$$\frac{d}{dt}\delta a(t) = \left(i\overline{\omega}_0 - \frac{\Gamma}{2}\right)\delta a(t) + iG\overline{a}\delta T(t) + \sqrt{\eta\Gamma}\delta s_{\rm in}(t) \tag{S6a}$$

$$\frac{d}{dt}\delta T(t) = \beta\left(\overline{a}^*\delta a(t) + \overline{a}\delta a^*(t)\right) - \gamma_{\rm th}\delta T(t) \tag{S6b}$$

We then make the following ansatz:

$$\delta a(t) = A_{\rm p}^- e^{+i(\omega_{\rm c}-\Omega)t} + A_{\rm p}^+ e^{+i(\omega_{\rm c}+\Omega)t}$$

$$\delta T(t) = Te^{-i\Omega t} + T^* e^{+i\Omega t}$$

where $A_{\rm p}^-$ and $A_{\rm p}^+$ represent the complex field amplitudes of respectively Stokes and anti-Stokes sidebands associated to the process. After including the latter into Eqs. (S6) and separating terms according to their time dependence, we finally derive the following solutions:

$$A_{\rm p}^- = \frac{iG\overline{a}T + \sqrt{\eta\Gamma}s_{\rm p}}{i\left(\overline{\Delta} - \Omega\right) + \Gamma/2} \tag{S7a}$$

$$A_{\rm p}^+ = \frac{iG\overline{a}}{i\left(\overline{\Delta} + \Omega\right) + \Gamma/2}T^* \tag{S7b}$$

$$T = \frac{\beta}{-i\Omega + \gamma_{\rm th}}\left[\overline{a}^* A_{\rm p}^- + \overline{a}(A_{\rm p}^+)^*\right] \tag{S7c}$$

S1.4 Analytic solutions for the field amplitudes

The Eqs. (S7) can be used to derive A_p^-, A_p^+ and T. In particular, the expression for the temperature oscillation reads[2]:

$$\boxed{T = \frac{\beta}{-i\Omega + \Gamma_\mathrm{TOIT}} \cdot \frac{\overline{a}^* \sqrt{\eta\Gamma}}{i\left(\overline{\Delta} - \Omega\right) + \Gamma/2} \cdot s_\mathrm{p}} \tag{S8}$$

where we defined:

$$\Gamma_\mathrm{TOIT} = \gamma_\mathrm{th} \left(1 + \frac{|\overline{a}|^2}{|\overline{a}_\mathrm{b}|^2} \tilde{\chi}\left(\overline{\Delta}\right)\right) \tag{S9}$$

Here, $|\overline{a}_\mathrm{b}|^2 = -\frac{K\Gamma}{2G\Gamma_\mathrm{abs}}$ represents the characteristic energy for optical bistability, already found in the expression of the steady state solution, while $\tilde{\chi}\left(\overline{\Delta}\right) = \frac{4\overline{\Delta}/\Gamma}{4\overline{\Delta}^2/\Gamma^2+1}$ is a thermo-optical response function[3], which can be interpreted as a susceptibility for the phenomenon.

The physical meaning of Eq. (S8) is the following: the term on the right-hand side of the product represents the optical response of the cavity under external excitation, while the term on the left-hand side, which is also a Lorentzian of width $2\Gamma_\mathrm{TOIT}$, represents the amplitude of thermal oscillation under an external driving. Remarkably, the width

---

[2]The explicit expression for T is derived as follows:

$$T = \frac{\beta}{-i\Omega + \gamma_\mathrm{th}} \left(\frac{iG|\overline{a}|^2}{i\left(\overline{\Delta} - \Omega\right) + \Gamma/2} T + \frac{-iG|\overline{a}|^2}{-i\left(\overline{\Delta} + \Omega\right) + \Gamma/2} T + \frac{\overline{a}^* \sqrt{\eta\Gamma} s_\mathrm{p}}{i\left(\overline{\Delta} - \Omega\right) + \Gamma/2}\right)$$

$$\Rightarrow T = \left(\frac{-i\Omega + \gamma_\mathrm{th}}{\beta} - \frac{iG|\overline{a}|^2}{i\left(\overline{\Delta} - \Omega\right) + \Gamma/2} - \frac{-iG|\overline{a}|^2}{-i\left(\overline{\Delta} + \Omega\right) + \Gamma/2}\right)^{-1} \frac{\overline{a}^* \sqrt{\eta\Gamma}}{i\left(\overline{\Delta} - \Omega\right) + \Gamma/2} s_\mathrm{p}$$

[3]The full expression for $\tilde{\chi}\left(\overline{\Delta}\right)$ is complex: $\tilde{\chi}\left(\overline{\Delta}\right) = \frac{4\overline{\Delta}/\Gamma}{4\left(\overline{\Delta}^2 - \Omega^2\right)/\Gamma^2 - i4\Omega/\Gamma + 1}$. The approximation assumes $|\Omega| \ll \Gamma/2$.

of the temperature oscillation curve increases in the blue-detuning regime ($\Gamma_{\mathrm{TOIT}} > \gamma_{\mathrm{th}}$) and decreases in the red-detuning regime ($0 < \Gamma_{\mathrm{TOIT}} < \gamma_{\mathrm{th}}$). In both cases, Γ_{TOIT} is either maximized or minimized for $\overline{\Delta} = \pm\Gamma/2$. In principle, it would also be possible to investigate the regime of intense pumping where $\Gamma_{\mathrm{TOIT}} < 0$: here, the peak visibility would progressively reduce while increasing the pumping energy, eventually encountering a second crossover from gain to absorption regime. However, this case is associated to an unstable solution of the thermo-optical bistability equations (S4), and it is not experimentally accessible. The investigation of this regime thus goes beyond the scope of this work, and we will thus assume $\Gamma_{\mathrm{TOIT}} > 0$ from now on.

Given the above expression, the A_{p}^{+} field can be analytically derived from Eq. (S7b):

$$A_{\mathrm{p}}^{+} \approx \frac{1}{i\Omega + \Gamma_{\mathrm{TOIT}}} \cdot \frac{iG\beta\overline{a}^2\sqrt{\eta\Gamma}}{\overline{\Delta}^2 - \Omega^2 + \Gamma^2/4} \cdot s_{\mathrm{p}}^{*} \tag{S10}$$

Again, the spectral lineshape of A_{p}^{+} is a Lorentzian, whose width depends directly on Γ_{TOIT}. Similar considerations as for the temperature oscillation apply.

A similar expression for the A_{p}^{-} field can be derived from Eq. (S7a). In this case, the coherent mixing between the input probe field and the shifted control field produces an asymmetric (Fano) lineshape.

Fig. S1 shows the predicted lineshape for the A_{p}^{-}, A_{p}^{+} and T fields, and their evolution as a function of the pump energy $|\overline{a}|^2$.

S1.5 Output power (optical beat)

Recalling the formalism of input-output relations (*1, 2*), we introduce the output field:

$$s_{\mathrm{out}}(t) = -\sqrt{\eta_{\mathrm{out}}\Gamma}\, a(t) \tag{S11}$$

Notice that there is no mixing between the input and output, and the coupling between the two channels is given only by the resonator (*resonant tunneling* configuration). More

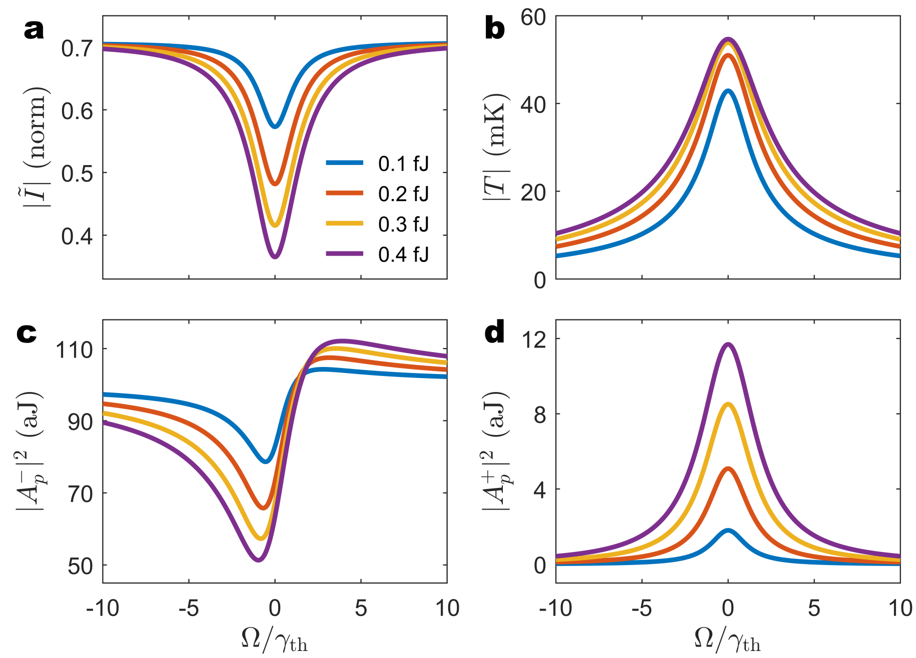

Figure S1: Predicted amplitude for the output signal (Eq. S12) and for the fields (Eq. S7) involved in the TOIT phenomenon as a function of the probe-control detuning. Each trace corresponds to a different value of control energy $|\overline{a}|^2$ and it is calculated at blue-detuning regime ($\overline{\Delta} = +\Gamma/2$). From top: **a.** output field amplitude (normalized to the cavity resonance) **b.** temperature oscillation amplitude **c.** energy of the anti-Stokes sideband and **d.** energy of the Stokes sideband.

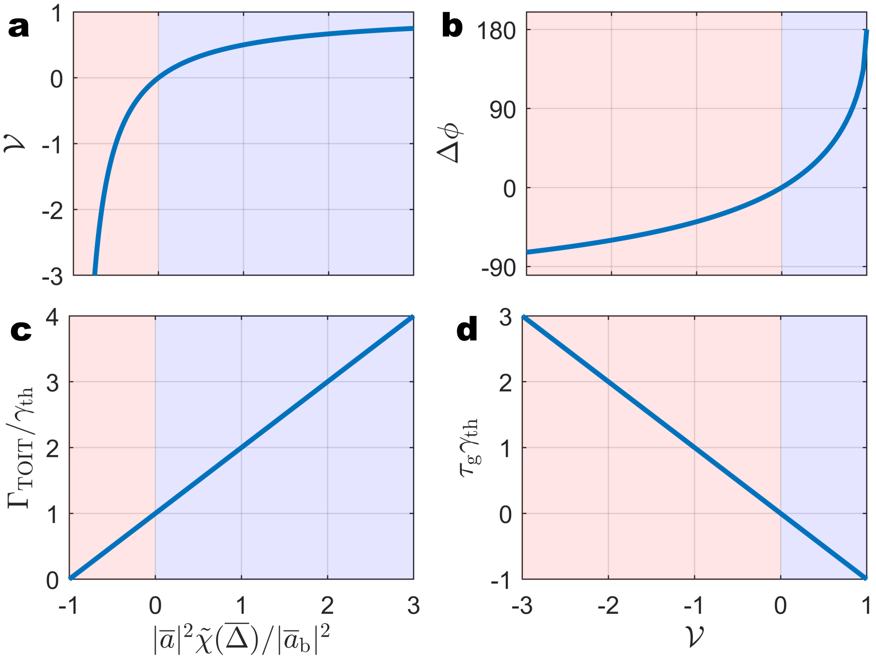

Figure S2: Predicted scaling trends for **a.** visibility, **b.** phase shift, **c.** induced trasparency linewidth and **d.** group delay from our theoretical model. Red (blue) shaded regions indicate a regime of positive (negative) control-cavity detuning $\overline{\Delta}$.

details about this configuration are provided in Section S3.3. For simplicity, we will assume symmetric in-out coupling, namely $\eta_{\text{in}} = \eta_{\text{out}} = \eta$, which effectively describes the resonant scattering (RS) experimental conditions[4].

Perhaps the most direct measurement scheme consists in the detection of the output field optical power $I(t) = |s_{\text{out}}(t)|^2$. Being characterized by the presence of three different frequency components, this exhibits interference in the time-domain (*optical beating*):

$$\begin{aligned} I(t) &= \eta\Gamma|\overline{a} + A_{\text{p}}^{-}e^{-i\Omega t} + A_{\text{p}}^{+}e^{+i\Omega t}|^2 \\ &= 2\eta\Gamma\Re\left\{\overline{a}^{*}A_{\text{p}}^{-}e^{-i\Omega t} + \overline{a}A_{\text{p}}^{+*}e^{-i\Omega t}\right\} + \ldots \\ &= 2\eta\Gamma\Re\left\{\overline{a}^{*}A_{\text{p}}^{-} + \overline{a}A_{\text{p}}^{+*}\right\}\cos\Omega t + 2\eta\Gamma\Im\left\{\overline{a}^{*}A_{\text{p}}^{-} + \overline{a}A_{\text{p}}^{+*}\right\}\sin\Omega t + \ldots \end{aligned}$$

where we omitted $(\ldots)$ the components that are constant in time. The last line provides a time-dependent expression for the output field oscillation. In order to better understand the physics of the process, we reformulate it in the frequency-dependent (complex) form: $\tilde{I}(\Omega) = 2\eta\Gamma\left(\overline{a}^{*}A_{\text{p}}^{-} + \overline{a}A_{\text{p}}^{+*}\right)$. From comparison with Eq. (S7c) and (S8), we can express:

$$\boxed{\tilde{I}(\Omega) = \left(1 - \frac{\Gamma_{\text{TOIT}} - \gamma_{\text{th}}}{-i\Omega + \Gamma_{\text{TOIT}}}\right) \cdot \frac{2\overline{a}^{*}\left(\eta\Gamma\right)^{3/2}}{i\left(\overline{\Delta} - \Omega\right) + \Gamma/2} \cdot s_{\text{p}}} \tag{S12}$$

In the blue (red) detuning regime, the multiplicative term within brackets represents a Lorentzian dip (peak) of width Γ_{TOIT} and visibility $|\mathcal{V}|$ such that:

$$\mathcal{V} = 1 - \frac{\gamma_{\text{th}}}{\Gamma_{\text{TOIT}}} \tag{S13}$$

This is a meaningful result, and it is reported in the main text. Notice that in this context

[4]Note that in this configuration, the highest coupling efficiency achievable is $\eta = 0.5$ (*5*). This is analogous, for instance, to the case of a symmetric Fabry-Pérot resonator, or a ring resonator in "add-drop" configuration.

we have defined the visibility as the height of the dip (peak) spectral feature divided by the value of the unperturbed cavity response[5] (i.e. in the absence of the TOIT phenomenon). The phase associated to expression (S12), defined such that the oscillating component of the output power takes the form $I(t) = |\tilde{I}| \cos(\Omega t + \phi)$, can be expressed as:

$$\phi(\Omega) = -\arg \tilde{I}(\Omega) \approx \arctan\left\{ \frac{+\Omega\left(\Gamma_{\text{TOIT}} - \gamma_{\text{th}}\right)}{\Omega^2 + \gamma_{\text{th}}\Gamma_{\text{TOIT}}} \right\} \tag{S14}$$

where we neglected the resonator phase response. The above function has absolute maximum and minimum in $\Omega = \pm\sqrt{\gamma_{\text{th}}\Gamma_{\text{TOIT}}}$, which corresponds to an overall phase-shift[6]:

$$\Delta\phi = 2\arctan\left\{ \frac{1}{2}\frac{\mathcal{V}}{\sqrt{1-\mathcal{V}}} \right\} \tag{S15}$$

Group delay is defined as $\tau_{\text{g}} = -\frac{d\phi}{d\Omega}$, and represents the temporal delay experienced by the electromagnetic field envelope $|a(t)|$. In the presence of optical beat, this can be expressed as:

$$\tau_{\text{g}}(\Omega) = -\frac{\phi}{\Omega} \overset{\Omega\to 0}{\approx} -\frac{\mathcal{V}}{\gamma_{\text{th}}} \tag{S16}$$

and exhibits a peak value for $\Omega \sim 0$. Notice that in the blue-detuning regime the delay is negative (group advance), and it asymptotically reaches the value $\tau_{\text{g}}^{\text{min}} = -1/\gamma_{\text{th}}$. In the red-detuning regime, the delay is positive as long as $\Gamma_{\text{TOIT}} > 0$, and it diverges on vanishing Γ_{TOIT}.

The predicted trends for $\mathcal{V}$, Γ_{TOIT}, $\Delta\phi$ and τ_{g} are shown in Fig. S2.

[5] Numerically and experimentally, the normalization is performed on the value of I at $\Gamma_{\text{TOIT}} \ll \Omega \ll \overline{\Delta}$.

[6] $\Delta\phi = [\max\phi(\Omega) - \min\phi(\Omega)] \cdot \text{sign}\,\mathcal{V}$.

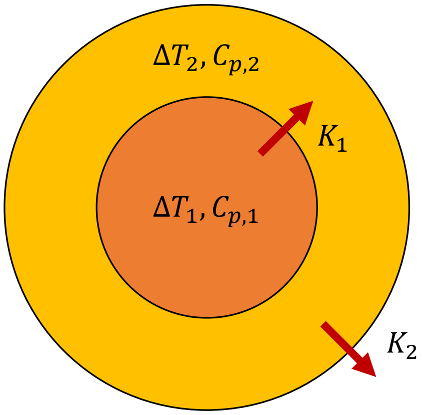

Figure S3: Discretized heat diffusion model. Each shell is characterized by a heat capacity $C_{p,i}$, and it exhibits a heat diffusion constant K_i and a temperature offset ΔT_i with respect to the next outer one.

S2 Modelling multiple thermal decay rates

The dynamic model presented in Sec. S1 and described by Eqs. (S1) approximates the thermal decay by a first order differential equation. Without any forcing term, the solution to Eq. (S2b) decays exponentially with a rate γ_{th}. This model represents a discretized version of the well known heat diffusion equation:

$$\rho c_p \frac{\partial(\Delta T)}{\partial t} + \nabla \cdot (-\kappa \nabla(\Delta T)) = \frac{\partial u}{\partial t} \tag{S17}$$

where ρ is the density of the material, c_p is the mass specific heat, κ is the thermal conductivity, and $\frac{\partial u}{\partial t}$ is a source term describing the heat flux density towards the system, in this case associated to the absorbed optical power.

On the other hand, the simplified formulation given by Eqs. (S1) may appear a too rough approximation for the detailed description of the actual system at hand. In fact, a more accurate and to some extent realistic description of the problem can be formulated

by a refined discretization, i.e., involving more than a single thermal decay rate, as also suggested by other authors (*4, 6*). As a consequence, we will henceforth consider 3 concentric regions (see, e.g., the scheme in Fig. S3), labeled from 1 to 3, such that $i = 1$ is the innermost one, where the field is mainly confined. The i-th region has a heat capacity $C_{p,i}$ an effective temperature difference ΔT_i with respect to the $(i+1)$-th one and a heat diffusion constant towards it K_i. Given these definitions, Eqs. (S2) are generalized as follows:

$$\frac{da(t)}{dt} = \left(i\omega_0 - \frac{\Gamma}{2}\right) a(t) + iG\Delta T(t)a(t) + \sqrt{\eta\Gamma}s_{\text{in}}(t) \tag{S18a}$$

$$\frac{d(\Delta T_1)}{dt} = \beta|a(t)|^2 - \gamma_{1,1}\Delta T_1(t) \tag{S18b}$$

$$\frac{d(\Delta T_2)}{dt} = \gamma_{1,2}\Delta T_1(t) - \gamma_{2,2}\Delta T_2(t) \tag{S18c}$$

$$\frac{d(\Delta T_3)}{dt} = \gamma_{2,3}\Delta T_2(t) - \gamma_{3,3}\Delta T_3(t) \tag{S18d}$$

where the effective temperature experienced by the field is $\Delta T = \Delta T_1 + \Delta T_2 + \Delta T_3$, while the rates connecting different regions are defined $\gamma_{i,j} = K_i/C_{p,j}$ and $\beta = \Gamma_{\text{abs}}/C_{p,1}$. With these definitions and the assumptions made in Sec. S1, Eqs. (S18) describe with high accuracy any thermal diffusion process that is consistent with the symmetry of the problem.

S2.1 Steady state solution

In the presence of a monochromatic pump field, a solution formally similar to Eq. (S4) can be found:

$$\overline{a} = \frac{\sqrt{\eta\Gamma}}{i\left(\omega - \omega_0 - G\overline{\Delta T}\right) + \Gamma/2}\overline{s}_{\text{in}} \tag{S19a}$$

$$\overline{\Delta T}_1 = \frac{\beta}{\gamma_{1,1}}|\overline{a}|^2 \tag{S19b}$$

$$\overline{\Delta T}_2 = \frac{K_1}{K_2}\overline{\Delta T}_1 \tag{S19c}$$

$$\overline{\Delta T}_3 = \frac{K_2}{K_3}\overline{\Delta T}_2 \tag{S19d}$$

In steady state, the diffusion constant K used in the simplified model takes here the role of a total thermal conductance, which can be expressed as $K^{-1} = K_1^{-1} + K_2^{-1} + K_3^{-1}$. The overall effective temperature offset can be then expressed as $\overline{\Delta T} = \frac{K_1}{K}\overline{\Delta T}_1$.

S2.2 Dynamical solution

Following the steps already discussed in Sec. S1, we will now derive the linearized equations of motion in the hypothesis of a small probe field. The procedure is formally identical to the previous one for what concerns the field amplitude, while for the effective temperature the following additional assumptions are made:

$$\Delta T_i(t) = \overline{\Delta T}_i + \delta T_i(t) \tag{S20a}$$

$$\delta T_i(t) = T_i e^{-i\Omega t} + T_i^* e^{+i\Omega t} \tag{S20b}$$

where:

$$\overline{\Delta T} = \sum_{i=1}^{3} \overline{\Delta T}_i \qquad \delta T = \sum_{i=1}^{3} \delta T_i \qquad T = \sum_{i=1}^{3} T_i$$

The linearized equations of motion (S18) thus read:

$$\frac{d}{dt}\delta a(t) = \left(i\overline{\omega}_0 - \frac{\Gamma}{2}\right)\delta a(t) + iG\overline{a}\delta T(t) + \sqrt{\eta\Gamma}\delta s_{\mathrm{in}}(t) \tag{S21a}$$

$$\frac{d}{dt}\delta T_1(t) = \beta\left(\overline{a}^*\delta a(t) + \overline{a}\delta a^*(t)\right) - \gamma_{1,1}\delta T_1(t) \tag{S21b}$$

$$\frac{d}{dt}\delta T_2(t) = \gamma_{1,2}\delta T_1(t) - \gamma_{2,2}\delta T_2(t) \tag{S21c}$$

$$\frac{d}{dt}\delta T_3(t) = \gamma_{2,3}\delta T_2(t) - \gamma_{3,3}\delta T_3(t) \tag{S21d}$$

We notice that the solution is formally similar to the one already found for a single thermal rate, Eqs. (S7), although the temperature oscillation T now derives from all the temperature offsets T_i:

$$A_{\mathrm{p}}^- = \frac{iG\overline{a}T + \sqrt{\eta\Gamma}s_{\mathrm{p}}}{i\left(\overline{\Delta} - \Omega\right) + \Gamma/2} \tag{S22a}$$

$$A_{\mathrm{p}}^+ = \frac{iG\overline{a}}{i\left(\overline{\Delta} + \Omega\right) + \Gamma/2}T^* \tag{S22b}$$

$$T_1 = \frac{\beta}{-i\Omega + \gamma_{1,1}}\left(\overline{a}^* A_{\mathrm{p}}^- + \overline{a}(A_{\mathrm{p}}^+)^*\right) \tag{S22c}$$

$$T_2 = \frac{\gamma_{1,2}}{-i\Omega + \gamma_{2,2}}T_1 \tag{S22d}$$

$$T_3 = \frac{\gamma_{2,3}}{-i\Omega + \gamma_{3,3}}T_2 \tag{S22e}$$

The total temperature oscillation is then given by:

$$T = \sum_{i=1}^{3} T_i = \underbrace{\left(1 + \frac{\gamma_{1,2}}{-i\Omega + \gamma_{2,2}} + \frac{\gamma_{1,2}}{-i\Omega + \gamma_{2,2}} \cdot \frac{\gamma_{2,3}}{-i\Omega + \gamma_{3,3}} + \right)}_{\xi(\Omega)} T_1 \tag{S23}$$

In the last step we defined the function $\xi(\Omega)$ such that $T = \xi(\Omega)T_1$. By directly comparing this with Eq. (S22c), we can derive the following analytic expressions for the generalized model:

$$T = \xi(\Omega)\frac{\beta}{-i\Omega + \Gamma_{\mathrm{TOIT}}(\Omega)} \cdot \frac{\overline{a}^* \sqrt{\eta\Gamma}}{i\left(\overline{\Delta} - \Omega\right) + \Gamma/2} \cdot s_{\mathrm{p}} \tag{S24}$$

$$\tilde{I} = \left(1 - \frac{\Gamma_{\mathrm{TOIT}}(\Omega) - \gamma_{1,1}}{-i\Omega + \Gamma_{\mathrm{TOIT}}(\Omega)}\right) \cdot \frac{2\overline{a}^* \left(\eta\Gamma\right)^{3/2}}{i\left(\overline{\Delta} - \Omega\right) + \Gamma/2} \cdot s_{\mathrm{p}} \tag{S25}$$

where:

$$\Gamma_{\mathrm{TOIT}} = \gamma_{1,1}\left(1 + \frac{|\overline{a}|^2}{|\overline{a}_{\mathrm{b}}'|^2}\xi(\Omega)\tilde{\chi}\left(\overline{\Delta}\right)\right) \tag{S26}$$

where $|\overline{a}_{\mathrm{b}}'|^2 = -\frac{K_1\Gamma}{2G\Gamma_{\mathrm{abs}}}$. Note that $\Gamma_{\mathrm{TOIT}}(\Omega)$ is now frequency-dependent, and loses here its physical interpretation as a linewidth. However, this quantity still provides useful information for the description of the phenomenon. In particular, we note that at low beating frequency $\xi(\Omega \to 0) = K_1/K$ and thus $|\overline{a}_{\mathrm{b}}'|^2/\xi(\Omega \to 0) = -\frac{K\Gamma}{2G\Gamma_{\mathrm{abs}}} = |\overline{a}_{\mathrm{b}}|^2$, which recovers a correspondence with the single-time case, Eq. (S9). Remarkably, the expression for visibility (S13) is substantially unvaried:

$$\mathcal{V} = 1 - \frac{\gamma_{1,1}}{\Gamma_{\mathrm{TOIT}}(0)} \tag{S27}$$

In contrast, the expressions for phase (S14) and group delay (S16) become here more

complicated. We will assume for the group delay as a function of $\mathcal{V}$ a trend similar to Eq. (S16):

$$\tau_{\mathrm{g}}(\Omega \to 0) = -\frac{\mathcal{V}}{\gamma_{\mathrm{th}}^{\mathrm{eff}}} \tag{S28}$$

where $\gamma_{\mathrm{th}}^{\mathrm{eff}}$ is an effective thermal decay rate, which is a function of the $\gamma_{i,j}$ parameters. In order to justify the (S28), we first divide $\xi(\Omega)$ into a real and an imaginary part:

$$\xi(\Omega) = \xi' + i\xi''$$

$$\Gamma_{\mathrm{TOIT}}(\Omega) = \gamma_{1,1} - \xi(\Omega)\zeta$$

where $\zeta = \frac{2G\beta|\overline{a}|^2\overline{\Delta}}{\overline{\Delta}^2+\Gamma^2/4}$. Then we express:

$$\tilde{I}(\Omega) = \frac{-i\Omega + \gamma_{1,1}}{-i\Omega\left(\Omega + \xi''\zeta\right) + \left(\gamma_{1,1} - \xi'\zeta\right)} \cdot \frac{2\overline{a}^*\left(\eta\Gamma\right)^{3/2}}{i\left(\overline{\Delta} - \Omega\right) + \Gamma/2} \cdot s_{\mathrm{p}}$$

from which we evaluate the phase:

$$\phi(\Omega) \approx \arctan\frac{\Omega\left(\gamma_{1,1} - \xi'\zeta\right) - \gamma_{1,1}\left(\Omega + \xi''\zeta\right)}{\Omega\left(\Omega + \xi''\zeta\right) + \gamma_{1,1}\left(\gamma_{1,1} - \xi'\zeta\right)}$$

where we neglected the bare resonator phase response. The asymptotic (negative) group delay can then be expressed as:

$$\tau_{\mathrm{g}} = -\lim_{\Omega\to 0}\frac{\phi}{\Omega} = -\frac{1}{\gamma_{1,1}}\left[1 - \frac{\gamma_{1,1}}{\Gamma_{\mathrm{TOIT}}}\left(1 + \xi''/\Omega\zeta\right)\right] \overset{\zeta\to\infty}{\approx} -\frac{1}{\xi'}\frac{d\xi''}{d\Omega} = \tau_{\mathrm{g}}^{\mathrm{min}}$$

where in the last step we assumed $\Gamma_{\mathrm{TOIT}}(0) \gg \gamma_{1,1}$ and thus $\mathcal{V} \to 1$ in the blue-detuning regime. The minimum group delay then takes the expression:

$$\tau_{\mathrm{g}}^{\mathrm{min}} = -\frac{1}{\gamma_{\mathrm{th}}^{\mathrm{eff}}} = -\frac{\gamma_{1,2}/\gamma_{2,2}^2 + \left(\gamma_{2,2} + \gamma_{3,3}\right)\gamma_{1,2}\gamma_{2,3}/\gamma_{2,2}^2\gamma_{3,3}^2}{1 + \gamma_{1,2}/\gamma_{2,2} + \gamma_{1,2}\gamma_{2,3}/\gamma_{2,2}\gamma_{3,3}}$$

S3 Experimental methods

S3.1 Sample design and fabrication

The dispersion adapted (DA) PhC cavity consists in a line defect along the ΓK direction of a triangular lattice of holes. The width of the line defect is gradually modulated with a quadratic profile by shifting the position of the innermost hole pairs along the transverse direction. Further details about the cavity design can be found at Ref. (*7*). In particular, we employed a sample with lattice period $a = 420\,\mathrm{nm}$, nominal radius $r = 120\,\mathrm{nm}$ and a predicted (finite-difference time-domain simulation) quality factor $> 10^7$. We modified this design by introducing a far-field optimization strategy (*8*) in order to improve the coupling to the Gaussian mode used for excitation along the normal direction with respect to the membrane plane.

The DA photonic crystal cavity was exposed in ZEP 520A resist by means of electron-beam lithography at 30 keV and the pattern transferred into the 220 nm thick silicon layer of an SOI wafer with a 2 µm thick buried oxide layer though dry etching in a fluorine-based (CHF_3/SF_6) plasma. The buried oxide layer underneath the PhC region was removed using a liquid hydrofluoric (HF) acid etch.

S3.2 Experimental setup

The experimental setup used to probe the TOIT phenomenon is schematically shown in Fig. S4. A continuous-wave tunable light source centered at 1550 nm (Santec TSL-710) is sent to a fiber-coupled beam-splitter (FBS). In the first output arm, light propagates unperturbed, while in the second a cascade of two acousto-optic modulators (AOM) first shifts the light frequency by −80 MHz and then by a variable amount between 55 MHz and 105 MHz. The two arms are then recombined by a second FBS, completing a Mach-Zehnder interferometer geometry. Both AOMs are coherently driven by a two-channel

arbitrary function generator (Tektronix AFG31152). The two AOMs are operated at the $m = \pm 1$ diffraction order in order to provide the correct sign to the frequency shift and to suppress any residual of the pump beam. The output signal so generated is thus composed of a pump field at frequency ω_{c} and a weak probe at frequency ω_{p}, with intensity ratio $10:1$, as verified by optical heterodyne measurements (see Sec. S5), and it is used to feed the resonant scattering (RS) apparatus.

This is essentially composed by a free-space cross-polarization spectroscopy arrangement (*5*), where the collimated light passes first through a linear polarizer (P) and then through a beam-splitter (BS) cube. The transmitted Gaussian beam is then focused on the sample by a microscope objective (MO) to a diffraction limited spot, the beam numerical aperture being optimized to match the one of the cavity mode. The sample is mounted on an aluminum holder, stabilized in temperature by means of a thermo-electric cooler module (TEC) and whose position is finely adjusted by piezo-electric positioners. The sample orientation is chosen to obtain a 45° orientation of the far-field polarization with respect to the one of the input beam. The output signal is collected along the same excitation path: the BS redirects part of it to a second linear polarizer (A) in order to suppress any spurious signal from the sample substrate, effectively isolating only the fraction of the field which is resonantly coupled to the cavity and backscattered in the crossed polarization.

The input and output signals of the RS setup are photodetected (PD) and amplified, and the resulting electrical signals are fed to a RF lock-in amplifier (Stanford Research Systems, model SR844), respectively as reference and signal input. The phase of the optical beat on the input signal is therefore taken as a reference for the output, thus providing an amplitude and phase readout for the quantity (S12).

The collected data is recorded and baseline corrected in order to compensate for the

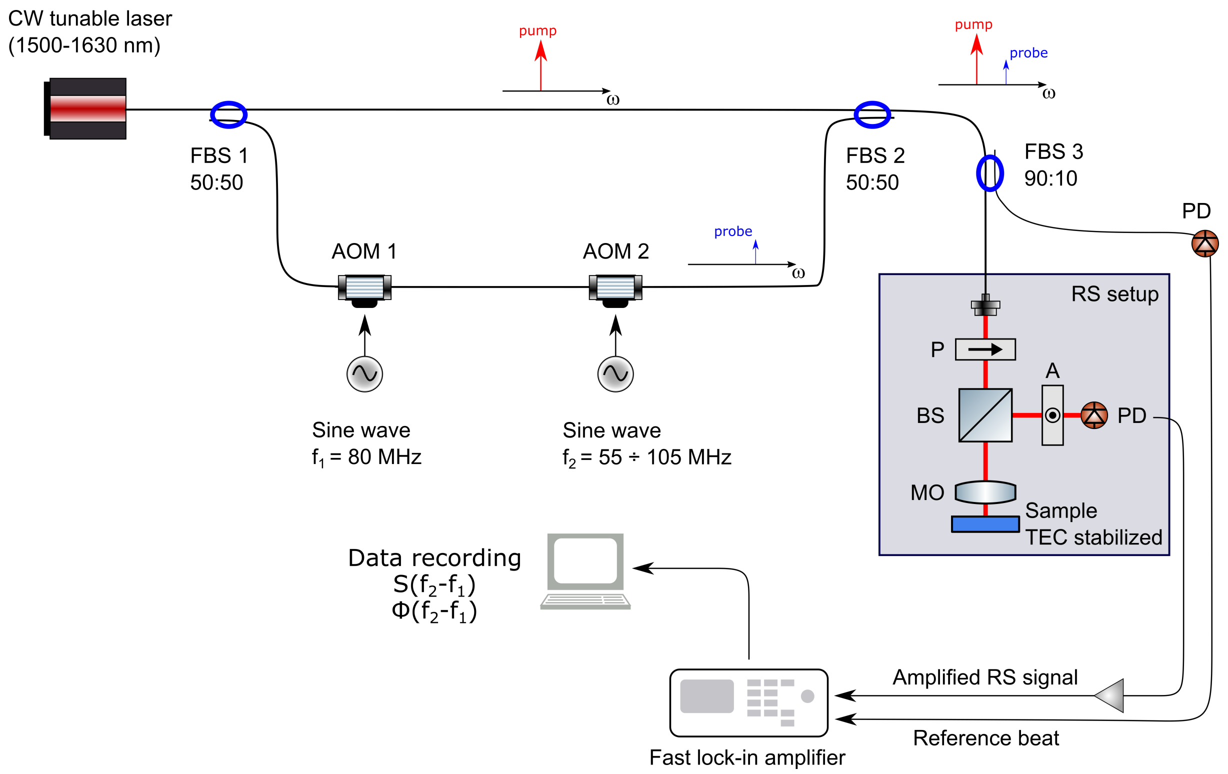

Figure S4: Experimental setup.

amplitude and phase response of the whole apparatus.

S3.3 Coupled-mode theory for the resonant scattering apparatus

Figure S5 shows a schematic of the input-output relations for the RS apparatus. In a simple coupled mode theory, consistent with Eqs. (S2), the cavity mode is represented by the field amplitude $a(t)$, it has resonance frequency ω_0 and total loss rate (linewidth) Γ. The incoming light consists of a Gaussian mode, of amplitude $s_{\rm in}(t)$, horizontally polarized, and it is coupled to the cavity mode with a rate $\Gamma_{\rm ext}^{\rm in}$. Similarly, the coupling with the output Gaussian mode $s_{\rm out}(t)$, vertically polarized, occurs at a rate $\Gamma_{\rm ext}^{\rm out}$. Note that the two channels (modes) are orthogonal and can thus be treated independently, in analogy with the *resonant tunneling* configuration.

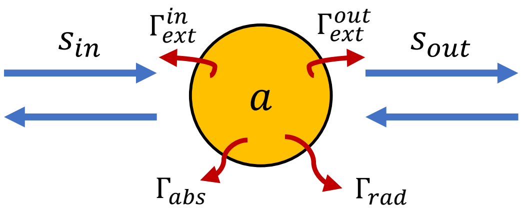

Figure S5: Coupled-mode schematic of the RS apparatus.

It is useful to introduce the in-coupling (out-coupling) efficiency $\eta_{\text{in}} = \Gamma_{\text{ext}}^{\text{in}}/\Gamma$ ($\eta_{\text{out}} = \Gamma_{\text{ext}}^{\text{out}}/\Gamma$), which represents the fraction of power which naturally decays to the input (output) mode in the absence of external excitation. Given the symmetric configuration (the cavity mode emission is linearly polarized and oriented at 45°), we assume $\eta_{\text{in}} = \eta_{\text{out}} = \eta$.

The remaining fraction of light may either be absorbed at a rate Γ_{abs}, giving origin to heating and thus thermo-optic shift, or be irradiated on modes different from the in- and out-coupling ones.

S3.4 Static sample characterization

S3.4.1 Linear spectroscopy

Linear spectroscopy was performed by feeding the RS apparatus with a tunable laser at low-power, in order to avoid a significant thermo-optic shift. The experimental spectrum obtained at $T = 300\,\text{K}$ is shown in Fig. S6a, for which we assessed a resonance frequency $\omega_0/2\pi = 193.61\,\text{THz}$ and a linewidth $\Gamma/2\pi = 5.09\,\text{GHz}$.

S3.4.2 Coupling efficiency

The coupling efficiency η was estimated by the following procedure. First, a RS spectrum is acquired with a known incident power P_{incident}. Light detection is performed in free-space, being sure that all the optical power is emitted within the objective field-of-view and collected. Then, the second polarizer (A) is aligned horizontally, the sample is replaced by

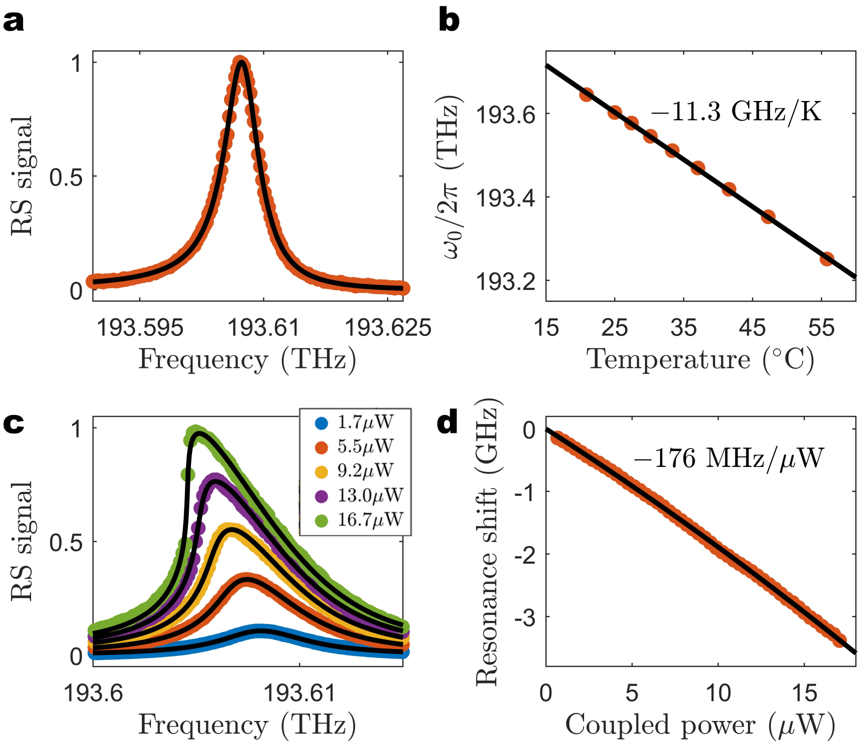

Figure S6: a. Resonant scattering spectrum of the PhC cavity used in this work, at low input power. **b.** Resonance frequency shift as a function of the sample temperature. **c.** Resonant scattering spectra obtained at increasing (from blue to green curves) incident power. Black curves are best fit obtained with model in Eq. (S5). Legend values indicate the estimated coupled power at the peak. **d.** Resonance shift as a function of the coupled power. Second-order polynomial fit (black solid curve) highlights a linear trend, associated to linear absorption, with a low quadratic contribution, associated to TPA and related nonlinear effects.

a high-reflectivity mirror and the measurement is repeated. The two values of collected power at the resonance wavelength are then compared as follows:

$$\eta_c = 8\frac{P_{\text{sample}}}{(1-\eta_{\text{abs}})P_{\text{mirror}}}$$

Here, $\eta_c = P_{\text{coupled}}/P_{\text{incident}}$ represents the estimated fraction of coupled power to the cavity in the best coupling condition (aligned polarization). The factor 8 accounts for the crossed polarization in input and output and for the cavity emission towards the substrate. From comparison with the coupled-mode theory described above, we derive $\eta = \eta_c/4$ in the best coupling conditions (incoming laser on-resonance, aligned polarizations). This value is further reduced by a factor 2 when the sample is excited in crossed-polarizations configuration.

The accurate estimate of η and incident power allows an accurate estimate of the intracavity energy $|\overline{a}|^2$ used in the TOIT experiment.

S3.4.3 Bistability trend

Fig. S6c shows the RS spectra obtained for increasing values of incident power. The characteristic sawtooth shaped spectral line is observed as predicted by Eqs. (S5). The model fit (solid lines) to the experimental spectra allow us to obtain the plot on the right-hand panel, namely the total thermo-optic shift as a function of the coupled power. The trend is approximately linear, with an only slight quadratic contribution: a signature of the fact that the heating mechanism is mainly owed to linear absorption phenomena. From a second-order polynomial fit of the trend, we obtain the coefficient $\xi = \frac{1}{\lambda_0}\frac{d\lambda_0}{dP_{\text{coupled}}}$. Finally, following Eq. (S5) the ratio $\eta_{\text{abs}} = \Gamma_{\text{abs}}/\Gamma$ can be evaluated as:

$$\eta_{\text{abs}} = \frac{K}{\lambda_0 \alpha} \cdot \frac{d\lambda_0}{dP_{\text{coupled}}} \approx 0.64$$

Note that, since the estimate of P_{coupled} depends on η_{abs}, the relation is recursive.

S3.4.4 Thermo-optic coefficient

The thermo-optic coefficient $\alpha = \frac{1}{\omega_0}\frac{d\omega_0}{dT}$ was estimated by studying the resonance shift as a function of the temperature externally set to the sample by the temperature controller. The results are shown in Fig. S6b, yielding a coefficient $\alpha = -5.85 \times 10^{-5}\,\text{K}^{-1}$, very close to the values reported in literature for dielectric modes in PhC cavities (*9*).

S3.4.5 Thermal properties

Fig. 1b (main text) shows the result of a finite element method (FEM) simulation of the PhC membrane temperature in stationary heating conditions. The temperature profile is notably much larger than the region where the optical mode localizes, thus justifying the effective temperature approximation ($\Delta T(x, y) \approx \Delta T$) both in the single- and multiple-thermal times models. From this simulation we extracted the thermal conductance $K = 3.9 \times 10^{-5}\,\text{W}\,\text{K}^{-1}$ and the heat capacity $C_p = 1.6 \times 10^{-11}\,\text{J}\,\text{K}^{-1}$.

S4 Switch-on measurements

In order to provide a further confirmation of the validity of the refined model described by Eqs. (S18), we investigated experimentally the dynamics of the thermo-optical response of the microresonator by temporally-resolved step-response measurements. The experimental configuration is shown in Fig. S7a: the RS apparatus was fed with the signal output from an electro-optic modulator (EOM) driven by a square wave signal (50 kHz, rise time

$< 2.5\,\mathrm{ns}$). The output signal from the sample was collected by a fast photodetector (bandwidth: 600 MHz) and recorded by an oscilloscope.

Fig. S7b shows the time-resolved RS signal associated to the thermo-optical response. Each colored trace corresponds to a different measurement acquired at fixed nominal power ($P = 10\,\mathrm{mW}$) and varying initial laser-cavity detuning condition. The traces are normalized to the peak value. Black solid lines represent the best-fit of the experimental data with the model described in Section S2. Specifically, in order to separate the (fast) dynamics of the optical electromagnetic field from the one of the thermal process, we re-arranged Eqs. (S18) in the following way:

$$\frac{d(\Delta T_1)}{dt} = \frac{4\eta\beta/\Gamma}{\left(\frac{\omega-\omega_0(1+\alpha\Delta T)}{\Gamma/2}\right)^2+1}|s_{\mathrm{in}}(t)|^2 - \gamma_{1,1}\Delta T_1(t) \tag{S29a}$$

$$\frac{d(\Delta T_i)}{dt} = \gamma_{i-1,i}\Delta T_{i-1}(t) - \gamma_{i,i}\Delta T_i(t) \qquad (i > 1) \tag{S29b}$$

These equations are formally identical to the result provided in Ref. (*4*) and can be derived from our model under the assumption that the thermal response is much slower than the optical one ($\gamma_{i,j} \ll \Gamma$). The fitting algorithm was operated by numerically integrating Eqs. (S29) at each iteration.

Model fit replicates very well the temporal dynamics, proving the validity of the model also in the framework of a step excitation. The thermal decay rates estimated are comparable, although underestimated with respect to the ones found in spectroscopic measurements, yielding the values: $\gamma_{1,1}/2\pi = 1.32\,\mathrm{MHz}$, $\gamma_{2,2}/2\pi = 0.24\,\mathrm{MHz}$ and $\gamma_{3,3}/2\pi = 0.11\,\mathrm{MHz}$. We believe that this discrepancy can be attributed to: 1) the large number of free parameters used for the fit and 2) the presence of nonlinear effects at high input power.

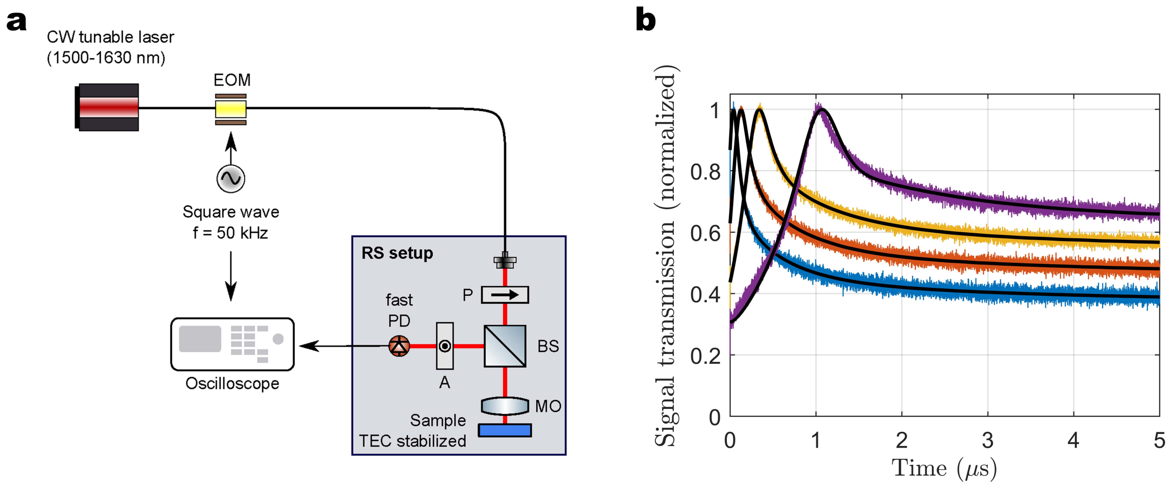

Figure S7: a. Experimental apparatus used for step-response measurements. **b.** Temporally resolved step-response.

S5 Optical heterodyne measurements

The observation of the measured output signal $\tilde{I}$ defined by Eq. (S12) represents a clear phenomenological signature of the undergoing physical process. However, the amplitude of this intensity oscillation depends on all the individual fields inside the cavity, and in particular on the sidebands A_p^- and A_p^+.

In order to provide a further confirmation of the undergoing phenomenon, we investigated the individual sidebands by an optical heterodyne detection scheme, as shown in Fig. S8. The experimental heterodyne spectra are shown in Fig. S9.

The anti-Stokes sideband A_p^+ (Fig. S9b,d) shows a clear Lorentzian-like lineshape, which is consistent with Eq. (S10) and the predicted spectrum represented in Fig. S1. Black solid curve in the blue-detuning regime represents the best fit of experimental spectrum with a double-Lorentzian lineshape, which is a natural lineshape guess in agreement with the multiple thermal rates model. This field can be viewed as a sideband originating from the modulation of the pump due to the thermo-optical oscillation.

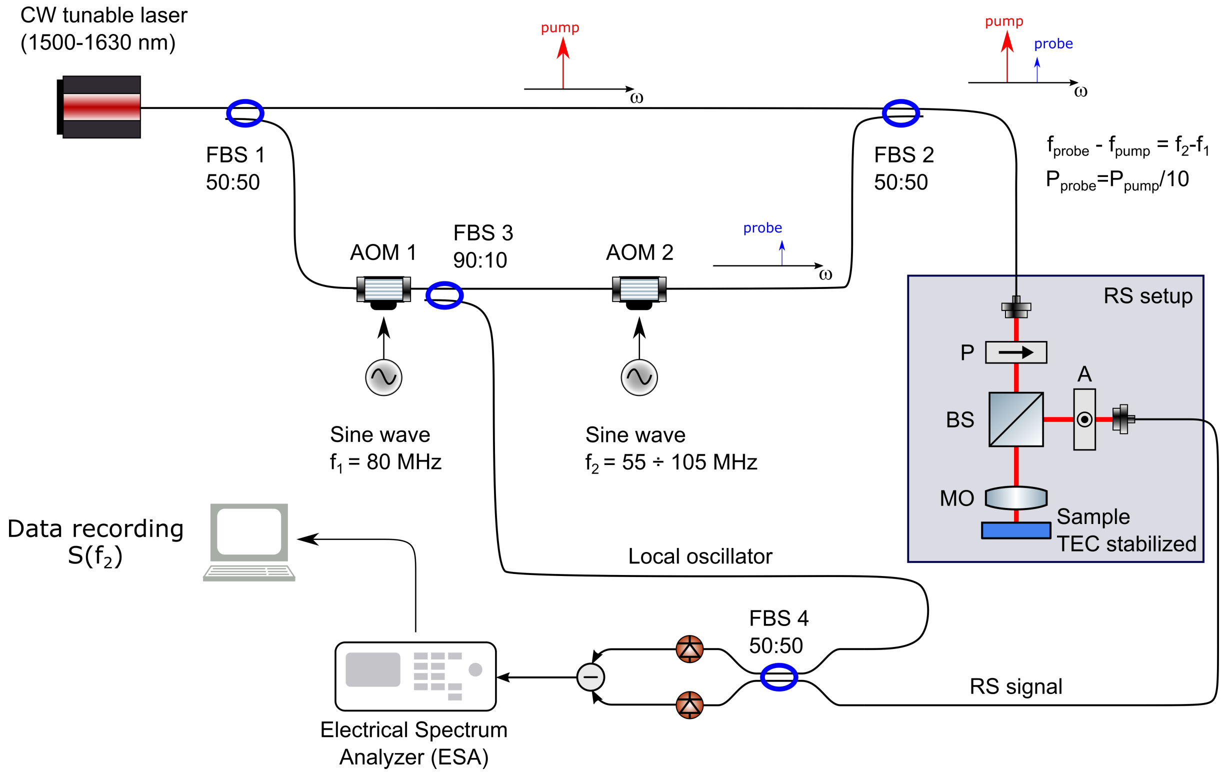

Figure S8: Experimental setup for optical heterodyne detection.

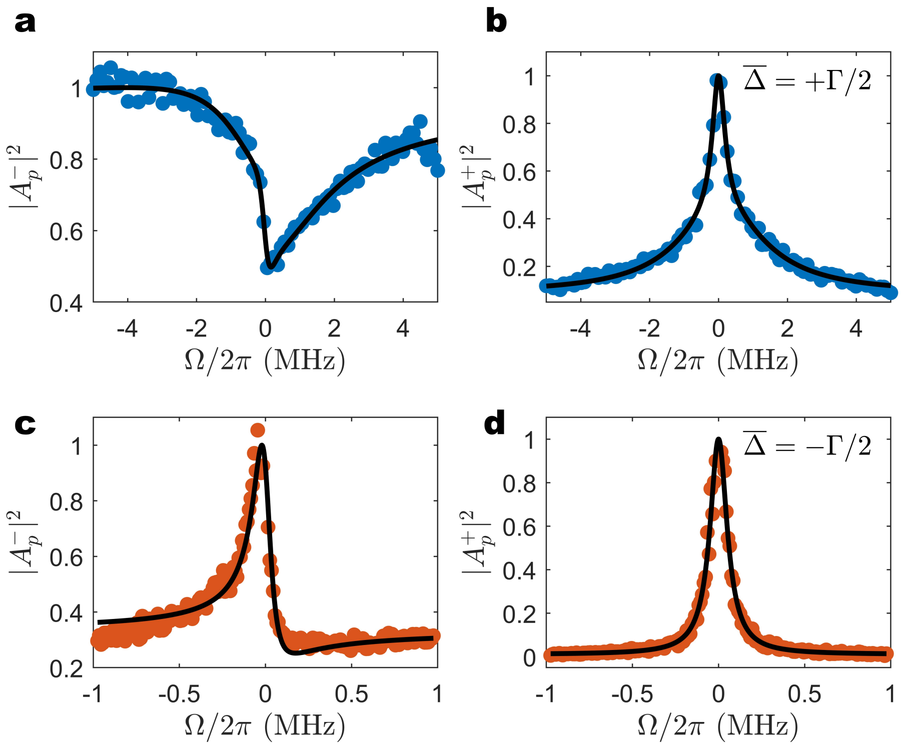

Figure S9: Optical heterodyne spectra (normalized) for the **a.** A_p^- and **b.** A_p^+ fields in the blue-detuning regime. Black solid curves are double-Fano and double-Lorentzian fits, respectively. **c-d.** The same spectra in the red-detuning regime are fitted with a single Fano and Lorentz model respectively.

Configuration	$\gamma_{1,1}/2\pi$	$\gamma_{1,2}/2\pi$	$\gamma_{2,2}/2\pi$	$\gamma_{2,3}/2\pi$	$\gamma_{3,3}/2\pi$
Si PhC - Ox 220nm	1.23	0.46	0.22	0.038	0.027
Si PhC - Si 220nm	6.40	4.15	0.80	0.002	0.038

Table S1: Thermal decay rates extracted from the fit of the simulated temperature decay curves (Fig. S10) with the 3-times analytical model for the two different cavity configurations (PhC laterally enclosed by silicon or silicon dioxide). All values are expressed in megahertz.

Similarly, the Stokes sideband A_{p}^{-} (Fig. S9a,c) shows a Fano lineshape, which can be attributed to the coherent interference between the input signal and the sideband produced by the probe modulation. The two fields combined exhibit Fano-type interference, and the black solid curve represents the best fit with the sum of two Fano lineshapes.

The linewidths (FWHM) derived from the fits in the blue-detuning regime are respectively: $2\Gamma_{\mathrm{TOIT}}^{(1)} = 3.2\,\mathrm{MHz}$ and $2\Gamma_{\mathrm{TOIT}}^{(2)} = 0.48\,\mathrm{MHz}$, consistently with the values reported in main text.

For the red-detuning regime, the fit was performed with a single Lorentzian or Fano lineshape and, owing to the narrowing of the spectral line, provided a smaller linewidth $2\Gamma_{\mathrm{TOIT}} = 0.12\,\mathrm{MHz}$.

S6 Engineering the thermal response

In this Section, we show the possibility to easily engineer the geometry of the cavity system in order to slow the diffusion of the heat generated by the optical pumping from the center of the PhC cavity to the rest of the structure. This translates into a slower cavity temperature decay in time and, as the thermal response of the system dominates the induced transparency, effectively corresponds to a narrowing of the TOIT peak/dip and related higher group delay or advance. Consequently, the time delay related to the TOIT practically achievable can potentially be increased by orders of magnitude just by tailoring the integrated cavity thermal insulation through the etching of opportune trenches,

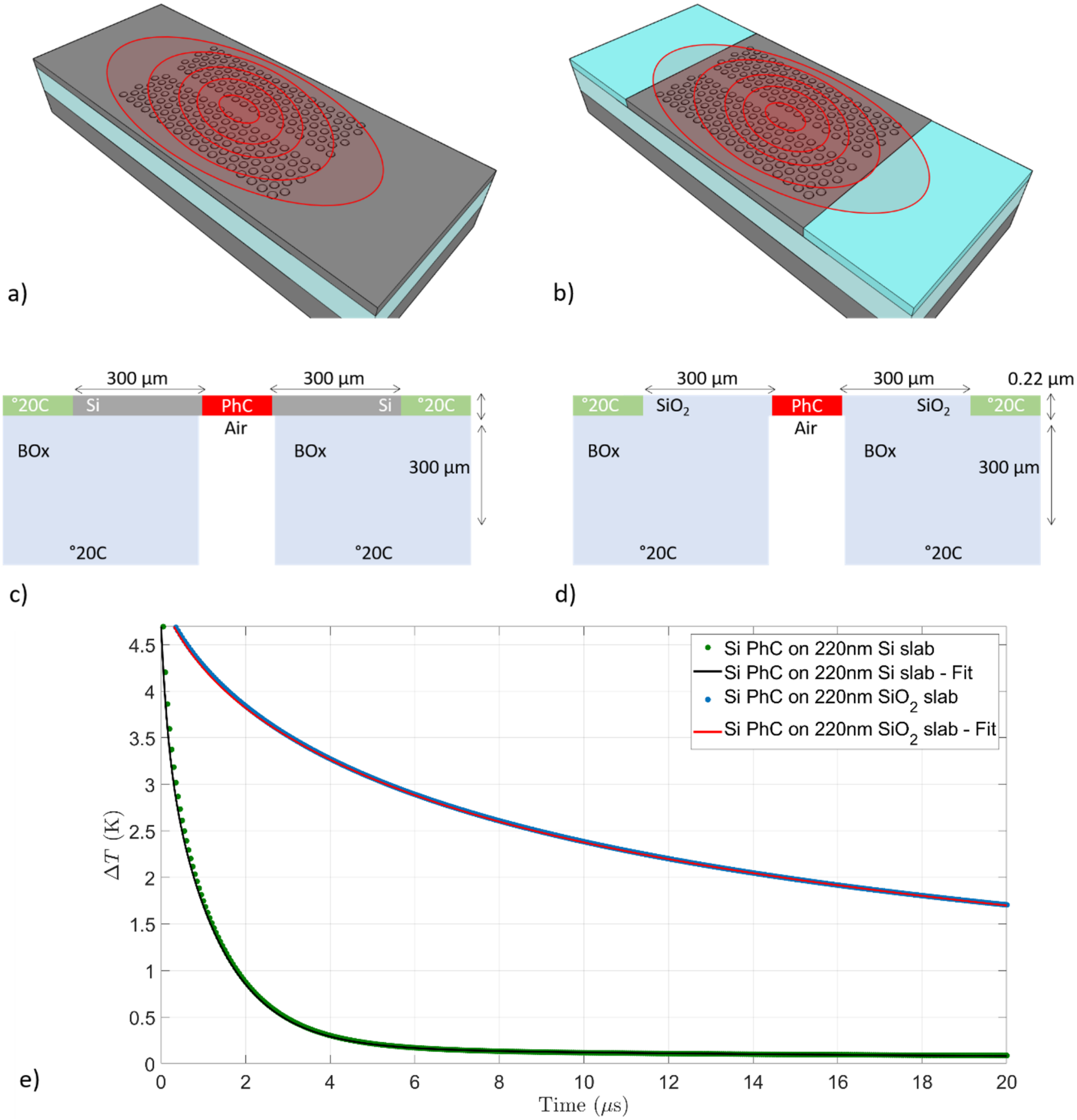

Figure S10: **a.** Schematics of the PhC cavity patterned on the SOI, **b.** schematics of the same PhC cavity with the lateral 220 nm thick Si replaced by silicon dioxide. Red ellipses represent conceptually the thermal volume expansion in time, as shown in Ref. (*10*), **c.** schematics of the cross-section of **a**, **d.** schematics of the cross-section of **b**, **e.** Temperature decay in time for the PhC in **a** (green dots) and **b** (blue dots) calculated with the numerical model in Ref. (*10*), and their respective fits with the 3-times analytical model (black and red curves).

undercutting the PhC membrane or the deposition of thermally insulating material such as silicon dioxide.

In Fig. S10a and Fig. S10b, schematics of two different PhC cavity geometries are shown. Fig. S10a represents an air-bridge PhC cavity patterned on a 220 nm thick silicon slab, supported by a 300 µm thick layer of buried oxide (BOx). This configuration has a similar geometry as the one used in the experimental measurement, the former only differing by having a thicker BOx. Fig. S10b represents the same air-bridge PhC cavity patterned on the SOI as the one in Fig. S10a, with the only difference that, in this configuration, the silicon slab laterally enclosing the cavity has been replaced by a 220 nm slab of silicon dioxide. Fig. S10c and Fig. S10d show the schematic cross-sections of the cavity geometries shown in Fig. S10a and Fig. S10b respectively. To estimate the different thermo-optic responses of the two geometries shown, we used the numerical model described in Ref. (*10*), setting all the materials at initial room temperature and considering a portion spanning 300 µm from each side of the PhC cavity. The temporally resolved step-response of the two optically pumped systems have been obtained numerically, and the temperature decays in time related to the different geometries have been plotted in Fig. S10e. These temperature decays are then fitted with the analytic model described by Eqs. S29, in order to extract the values of the thermal decay rates required for the calculation of the group delay in the two different cases. From Fig. S10e it is clearly noticeable how the configuration with lateral silicon dioxide yields a more than one order of magnitude slower temperature decay compared to the one with case with silicon surroundings. The corresponding thermal decay rates are shown in Table S1.

From the fit of the numerically computed thermal decay rates of the two different cavity configurations, we calculate a group delay $\tau_g = 1.76\,\mu s$ (at $\mathcal{V} = -1$) for the PhC cavity surrounded by silicon and a group delay $\tau_g = 22.5\,\mu s$ (at $\mathcal{V} = -1$) for the same

cavity laterally surrounded by silicon dioxide, leading to a >10-fold increase of the group delay achievable at the same peak visibility by simply tailoring the PhC cavity thermal insulation.

S7 Measurements dataset

Here we report, for completeness, the full dataset of TOIT amplitude and phase measurements. The experimental scans are presented as intensity plots in Fig. S11. Each intensity plot is associated to a different value of nominal control power.

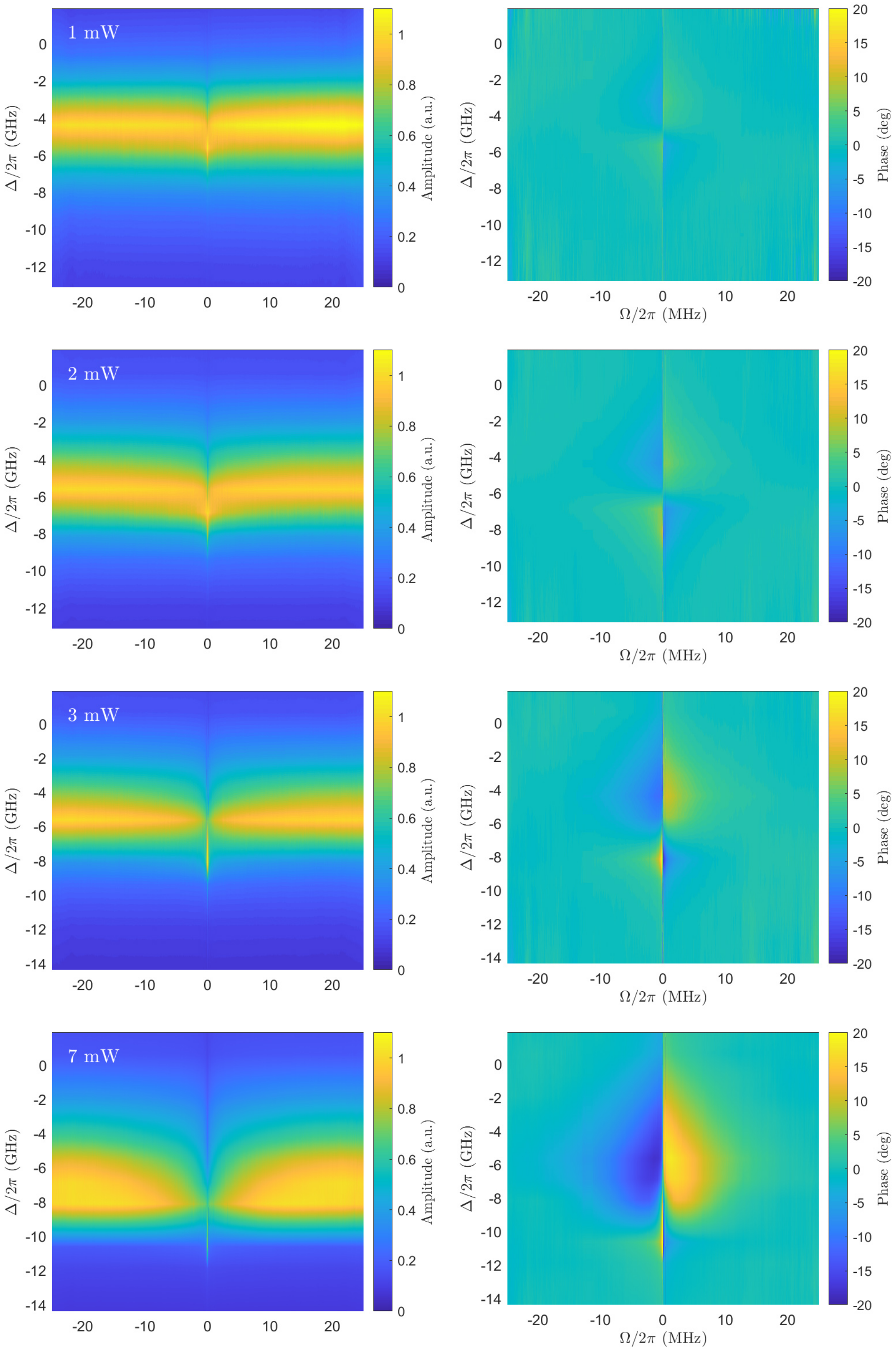

Figure S11: Color maps of the measured amplitude (*left*) and phase (*right*) as a function of the control-probe detuning frequency Ω and control-cavity detuning $\Delta = \omega_c - \omega_0$ for different values of nominal input power. The measurements complement the dataset presented in the main text.

References

1. Joannopoulos, J. D., Johnson, S. G., Winn, J. N. & Meade, R. D. *Photonic Crystals: Molding the Flow of Light* (Princeton University Press, 41 William Street Princeton, NJ, USA, 2008).

2. Aspelmeyer, M., Kippenberg, T. J. & Marquardt, F. Cavity optomechanics. *Reviews of Modern Physics* **86**, 1391–1452 (2014). `0712.1618`.

3. Soljačić, M., Ibanescu, M., Johnson, S. G., Fink, Y. & Joannopoulos, J. D. Optimal bistable switching in nonlinear photonic crystals. *Phys. Rev. E* **66**, 055601 (2002).

4. Carmon, T., Yang, L. & Vahala, K. J. Dynamical thermal behavior and thermal self-stability of microcavities. *Opt. Express* **12**, 4742 (2004).

5. Galli, M. *et al.* Light scattering and Fano resonances in high-Q photonic crystal nanocavities. *Applied Physics Letters* **94**, 071101 (2009).

6. Ilchenko, V. & Gorodetsky, M. Thermal nonlinear effects in optical whispering gallery microresonators. *Laser Phys.* **2**, 1004–1009 (1992).

7. Welna, K., Portalupi, S. L., Galli, M., O'Faolain, L. & Krauss, T. F. Novel Dispersion-Adapted Photonic Crystal Cavity With Improved Disorder Stability. *IEEE J. Quantum Electron.* **48**, 1177–1183 (2012).

8. Portalupi, S. L. *et al.* Planar photonic crystal cavities with far-field optimization for high coupling efficiency and quality factor. *Opt. Express* **18**, 16064 (2010).

9. Barclay, P. E., Srinivasan, K. & Painter, O. Nonlinear response of silicon photonic crystal micresonators excited via an integrated waveguide and fiber taper. *Opt. Express* **13**, 801 (2005).

10. Iadanza, S. *et al.* Model of thermo-optic nonlinear dynamics of photonic crystal cavities. *Phys. Rev. B* **102**, 245404 (2020).
